# Biosphere Reserve for All: Potentials for Involving Underrepresented Age Groups in the Development of a Biosphere Reserve through Intergenerational Practice

**DOI:** 10.1007/s00267-018-1059-9

**Published:** 2018-05-22

**Authors:** Tamara Mitrofanenko, Julia Snajdr, Andreas Muhar, Marianne Penker, Elisabeth Schauppenlehner-Kloyber

**Affiliations:** 1 Institute of Landscape Development Recreation and Conservation Planning, University of Natural Resources and Life Sciences, Vienna, Austria; 2 Master graduate from the University of Natural Resources and Life Sciences, Vienna, Austria; 3 Institute for Sustainable Economic Development, University of Natural Resources and Life Sciences, Vienna, Austria

**Keywords:** Biosphere reserve, Participation, Intergenerational practice, Youth, Elderly women

## Abstract

Stakeholder participation is of high importance in UNESCO biosphere reserves as model regions for sustainable development; however, certain groups remain underrepresented. The paper proposes Intergenerational Practice (IP) as a means of involving youth and elderly women and explores its options and barriers, using the example of the Salzburger Lungau and Kärntner Nockberge Biosphere Reserve in Austria. Case study analysis is used involving mixed methods. The results reveal obstacles and motivations to participating in biosphere reserve implementation and intergenerational activities for the youth and the elderly women and imply that much potential for IP exists in the biosphere reserve region. The authors propose suitable solutions from the intergenerational field to overcome identified participation obstacles and suggest benefits of incorporating IP as a management tool into biosphere reserve activities. Suggestions for future research include evaluating applications of IP in the context of protected areas, testing of methods used in other contexts, and contribution to theory development.

## Introduction

### Biosphere Reserves and Participation

Biosphere reserves are conceptualized by the UNESCO Man and the Biosphere Program (MAB) as model regions for sustainable development, where the paradigm of combining nature conservation with economic development and maintaining cultural values is tested, refined and implemented (UNESCO 1996). The recent MAB strategy 2015–2025 envisages an even stronger role for the biosphere reserves in reconciling conservation with human needs. It positions them as the “principal internationally designated areas dedicated to sustainable development in the twenty-first century” (UNESCO 2017, 15) and explicitly refers to their contribution to the global *Sustainable Development Goals*, adopted by the United Nations in 2015 as part of the *2030 Agenda for Sustainable Development* (UN 2015, Köck and Arnberger, 2017). Involvement of the local population is a key element of the biosphere reserve concept, and thus is also highlighted in the objectives outlined in the Seville Strategy (UNESCO 1996; Stoll-Kleemann et al. 2010). Literature on the subject advocates for all-encompassing participation in all management aspects: “in defining objectives, choosing between alternative courses of action, implementation, and, finally, evaluation” (Stoll-Kleemann and Welp 2008, p 162). The current MAB strategy calls for even stronger participation and integration of the local population and their economic activities in biosphere reserve policy and management (“selecting, designating, planning, and implementing”) (UNESCO 2017, p 22), thus “enabling people to become pioneers and ambassadors for realizing effective sustainability in all Biosphere Reserves” (Stoll-Kleemann and O’Riordan 2017, p 89).

Participation is defined as “a process where individuals, groups and organizations choose to take an active role in making decisions that affect them” (Reed 2008, p 2418). Involvement of diverse population groups in local planning and development processes, including conservation activities, is a critical issue of both equity and environmental justice (Zeldin et al. 2005; Buffel et al. 2014; Mitrofanenko 2016) and a democratic necessity (Schliep and Stoll-Kleemann 2010). It can facilitate a local sense of place and sustainable community development (Edge and McAllister 2009; Holden 2011) and ensure long-term success and quality in particular of those management processes, which require integration of different forms of knowledge and co-management (Zeldin et al. 2005; Berkes 2009; Raymond et al. 2010; Schliep and Stoll-Kleemann 2010; Schauppenlehner-Kloyber and Penker 2014). In the case of protected areas, understanding their objectives and the rationale for their designation is a major factor in developing a positive attitude towards these areas among the local population (Xu et al. 2006; Huber and Arnberger 2016; Hernes and Metzger 2017; Van Cuong et al. 2017); besides, participatory management approaches may facilitate a higher degree of legitimacy and acceptance of processes and solutions by creating local support for protected area-related decisions and management practices after designation (Stoll-Kleemann and Welp 2008).

Multiple studies have determined various motivations for participation in local events and processes. These include acceptance of the purpose, feelings of ownership and making a contribution, possibilities of empowerment, equity, trust and learning, social links and networks, trust in public authorities, a personal invitation to an event or material compensation (Ravindra 2004; Reed 2008; Pickering Sherman et al. 2010; Enengel et al. 2011; Huber 2011; Davis et al. 2012). At the same time, a number of obstacles to participation have been identified in literature: perception of power inequality, inability to influence decision-making, unequal representation of stakeholders with respect to age, gender, and social background, lack of trust and agreement, lack of interest and incentives, lack of confidence, time and financial resources, low mobility, consultation fatigue, as well as lack of information (Ravindra 2004; Reed 2008; Ianni et al. 2010; Huber 2011; Lupou et al. 2010; Enengel et al. 2011; Méndez-López et al. 2015). Due to these and other obstacles, certain groups remain underrepresented in sustainable development processes. With respect to gender, women have been historically underrepresented in decision-making. However, the importance of their involvement in all spheres, including nature protection, has been addressed by a number of studies, as well as strategies and policy documents (Cornwall 2003; Martino 2008; Khadka and Verma 2012; UN 2012; Schmitt 2014).

With respect to age groups, the older and the younger people “are more likely than other groups to lack access to decision-making channels, and also to lack political representation and to participate less in public life” (Pain 2005 in Buffel et al. 2014, p 1788). The lack of participation among the elderly and the youth in processes related to sustainable community development, such as biosphere reserve management, not only undermines equity and fairness, but also prevents inclusion of their experiences and ideas, which could affect the quality of results and the success of decisions in the long-term. Moreover, involvement of the youth and the elderly and interaction among them constitute important elements of sustainable development, which is grounded in connections among the present and future generations and draws from awareness of cultural and natural heritage (UN 2013; Mitrofanenko 2016). For example, it can ensure that the younger generation preserves traditional and local knowledge^1^ by adapting it to new problems or by applying new knowledge and skills, such as Information Technology.

The importance of considering the needs of the young and elderly people and of their engagement is recognized in international documents relevant to biosphere reserves, such as Agenda 21 (UN 1992, para 7.77, 10.10, and 7.4, respectively), which the Seville Strategy aims to help implement. Agenda 21 acknowledges youth among the groups often excluded from decision-making, along with women and indigenous groups. The MAB strategy for 2015–2025 explicitly refers to consideration of young people in “equitable and participatory planning for sustainable development in biosphere” (UNESCO 2017, p 18). Albeit less directly, the contribution of the elderly people to provision of the traditional and local knowledge could be inferred from the text of the MAB strategy (i.e., Strategic Objective 3, UNESCO 2017, p 19). Moreover, the *2030 Agenda for Sustainable Development*, to which the strategy aims to contribute, calls for empowerment of youth and older people among the other vulnerable groups (UN 2015, para 23).

Potential knowledge-, attitude- and action-related contributions from the older and younger generations to protected area management have been presented by Mitrofanenko et al. (2015). However, in addition to the younger or the older generations’ contribution, protected areas can also benefit from Intergenerational Practice (IP), i.e., the interaction of the two generations.

### Intergenerational Practice

Intergenerational Practice “aims to bring people together in purposeful, mutually beneficial activities, which promote greater understanding and respect between generations and may contribute to building more cohesive communities” (EAGLE Project 2008, p 5). Terms, such as Intergenerational Programming (Alcock et al. 2011) or Intergenerational Programs (Newman and Hatton-Yeo 2008), are also used, although a more recent publication refers to “practice” as a broader realm and names several other labels used in various disciplines to describe the “intergenerational engagement phenomena.” The authors position intergenerational practice as an initial component of the developing intergenerational field complimented by theory and policy and advocate the need for research to facilitate further understanding of this interdisciplinary research area (Bernard 2006; Kaplan and Sanchez 2014).

In fact, a number of details remain ambiguous in intergenerational literature, which is rather typical of an emerging field founded on practice rather than theory (Newman et al. 1997). While multigenerational interactions are sometimes mentioned, most literature involves interaction among the younger (children and youth) and older people. Many authors also prioritize intergenerational interventions in an extra-familiar context to those within families. This is due to the roots of intergenerational practice, grounded in social intervention programs aimed at reinforcing links between the youngest and oldest generations (especially in conditions when familial relations between them are weakened). These links are substantiated by developmental psychology, which suggests particular complementarity between these age cohorts (Newman et al. 1997). Nevertheless, based on a profound review of literature on IP, Springate et al. (2008) call for greater clarity with respect to distinction between multi/intergenerational approaches.

The age of younger and older participants also varies among the authors, and a standard definition is lacking: young people are defined as those under 25, according to some studies (Springate et al. 2008), or under 20, according to others (Kaplan et al. 1998 in Boström 2003), while the age of older people ranges from ‘‘over 50’’ to ‘‘over 60’’ (Springate et al. 2008). Newman et al. distinguish among the ‘‘young-old’’ (65–75 years old), ‘‘old-old’’ (75–90) and ‘‘very old’’ (90 and older) adults (1997). The UN uses the following definition for statistical purposes: the ‘‘youth’’ are persons between the ages of 15–24 “without prejudice to other definitions by Member States” (UN 2001, para 3), while the term ‘‘young people’’ includes both ‘‘youth’’ and ‘‘adolescents’’ (10–19 years old); alterative age ranges are adopted by several UN entities (Karkara et al. 2012). While UN does not provide a definition of the term ‘‘older’’, ‘‘60+’’ has been used in various UN documents (Kowal and Dowd 2001). The UN report on World Population Ageing makes a distinction between ‘‘older persons’’ (60 and over) and the ‘‘oldest-old’’ (80 and over) (UNDESA 2015). The definition adopted in our study is in line with those described above: ‘‘60 and over’’ for the older, and ‘‘20 or under’’ for the younger participants, keeping in mind the official Austrian definition of youth (aged 14 to 19 years) (Großegger, 2003). The reason for the lack of clear definitions in existing literature might be the different understanding of age among cultures and nations, as well as the changing “political, economic and socio-cultural circumstances,” including the changing life expectancy (Newman et al. 1997; UN 2001, para 3).

Originally a response to the rapid demographic change, shifting family structures and a perception of growing distance between the generations in Europe, IP has been broadened in scope to facilitate community revitalization, integration of immigrants, active ageing and social inclusion (Sanchez et al. 2008; Springate et al. 2008; Buffel et al. 2014; Hatton-Yeo 2014). Buffel et al. (2014) list four key features of effective IP: (1) providing opportunities for the development of relationships between generations; (2) access to a range of support mechanisms (e.g., organizational support, community support); (3) providing opportunities for generations to do a range of things together; (4) taking account of program-specific issues, such as gender, culture, and language.

### Implementing IP in Biosphere Reserves

Mitrofanenko et al. (2015) have introduced Intergenerational Practice (IP) as an approach to involving young and old age groups in protected area management. They proposed IP-based solution pathways for specific protected area management challenges ranging from a single protected area to the international level. The authors pointed to the lack of empirical insights into the applicability of this approach in concrete protected areas. They also proposed that applying IP could be particularly promising in the case of biosphere reserves due to their specific role as test-beds for social innovation. While a “long-term, intergenerational perspective” is mentioned in the biosphere reserve concept (UNESCO 1996), scientific literature referring to intergenerational interactions in biosphere reserves—or protected areas in general—is lacking.

### Objectives

This article aims to fill the empirical gap by exploring the potential of IP for encouraging participation of the younger and elderly population in biosphere reserve implementation. We envision that participation of these groups could be especially relevant for, although by no means limited to, activities contributing to the strategic objectives 2—contribution to building sustainable societies, and 3—science, education and capacity building—of the current MAB strategy 2015–2025. We address the obstacles in representation of youth and elderly women in protected area processes and examine intergenerational practice as a possible tool for supporting their participation. The paper draws on insights from the Salzburger Lungau and Kärntner Nockberge Biosphere Reserve in Austria as a test case.

## The Case Study

The ‘‘Biosphere Reserve Salzburger Lungau and Kärntner Nockberge’’ was only recognized by UNESCO in 2012 and thus serves as a promising case study, as its recent history allows reflecting on its development process from the beginning. Moreover, it is possible to compare the applicability of IP in two different contexts as the biosphere reserve was set-up independently and distinctly in two Austrian provinces, and the efforts were joined only at the last moment (Fanninger 2012).

### Description of the Region

**Fig. 1:**
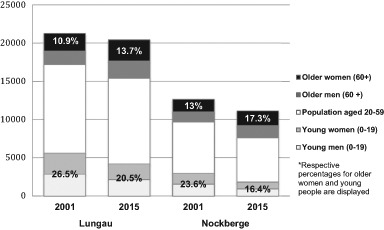
Population Composition of the Lungau and Nockberge Biosphere Reserve Region by Age and Gender (Statistik Austria 2017a; Kurz 2015)

The Biosphärenpark Salzburger Lungau and Kärntner Nockberge^2^ is the biggest biosphere reserve in Austria. It covers parts of two federal provinces: Salzburg and Carinthia. The alpine biosphere reserve is characterized by a diverse patchwork of cultural and natural landscapes, a high range of altitudes and a variety of traditional land uses (Huber et al. 2014). Youth (under 20 years old) constitute approximately 20 percent and elderly women (60 and older)—approximately 15 percent of the population of the biosphere reserve region, which reflects the Austrian average (Statistik Austria 2017,3). Figure 1 illustrates the share of youth and elderly women among the population in the Nockberge and Lungau provinces encompassing the reserve in 2001 and 2015.

At the time of the nomination for the biosphere reserve, the local communities in both provinces were characterized by aging and outmigration of the young population as well as relatively high unemployment and population loss rates (Regionalverband Lungau 2011; Huber 2011; Huber et al. 2014; Nigmann et al 2016). The decline of the younger population and increase of the elderly are also evident in Fig. 1.^4^,^5^ In both provinces, the local population was consulted regarding the potential biosphere reserve, and various media sources and events have been used to inform and involve the public (Jungmeier et al. 2009; Fanninger 2012; Huber et al. 2014; Huber and Arnberger 2016). While finally being designated as one biosphere reserve, the initial motivations and pathways towards the formal designation evolved independently and differed between the two provinces.

One of the motivations of the local communities in Lungau for the biosphere reserve designation was to better utilize the tourism potential of the region (Fanninger 2012). The results of a survey conducted among the local population shortly before the biosphere reserve designation in 2012 indicated positive attitudes towards the biosphere reserve, while at the same time, the majority of respondents reported being insufficiently informed about the biosphere reserve (Huber and Arnberger 2016).

In the case of the Carinthian Nockberge, it encompasses the territory of a former national park, which had failed to receive IUCN recognition and was redeveloped as a biosphere reserve with some consultation of the local population (Jungmeier et al. 2009). The National Park managers became biosphere reserve managers and could rely on well-established procedures and infrastructure.

According to the accounts of three biosphere reserve managers and two process facilitators^6^, who have accompanied all meetings and events in each province, as well as to findings of previous studies (i.e., Huber et al. 2014), the younger people and the elderly women were the least represented groups involved in setting up the reserve in both parts. Recently, school projects have been successfully implemented, co-organized by the Biosphere Reserve. They included excursions in the reserve and workshops, integrated into the school courses, as well as joint material development with the teachers. Local schools were renamed into Biosphere Reserve Schools to highlight the important link between education and the biosphere reserve. Despite these efforts, the managers of both parts encounter continued obstacles to participation of young people and even more so of elderly women.

### Specific Research Questions

The paper examines the motivations and barriers for participation of the youth and elderly women in processes and activities related to the implementation of the biosphere reserve and considers the potential for applying IP as a means to increase their involvement by addressing the following questions:

1. What are the perceptions of the biosphere reserve among the elderly women and youth, as well as the obstacles to and motivations for them to engage in biosphere reserve-related activities?
2. What are the elderly and younger residents’ perceptions of the other generation and intergenerational practice?
3. Are the biosphere reserve managers aware of the potential held by the involvement of youth and elderly, and to which extent are they familiar with IP?

Based on the analysis of our findings and corresponding results from literature on IP, in the final section we propose solutions that could facilitate better involvement of the young and elderly population in this case, but also in other biosphere reserves and protected areas.

## Methods

We used mixed methods, such as semi-structured interviews, focus groups and World Café (Kuckartz 2014), for data collection to best address respective groups of interviewees. The purposive sampling technique and key-informant interviews (Tongco 2007) helped collect data from the biosphere reserve managers and process facilitators in order to receive background information as well as references to other interviewees. In addition to face-to-face interviews with individual young residents, the World café method was applied in school classes in order to provide a bigger group of youth with an atmosphere free of hierarchy and to facilitate reflection-oriented discussion among the young participants. Snowball sampling was used to identify elderly women interviewees: initial contacts, some of them recommended by the key informants, referred the researchers to further contacts (Atkinson and Flint 2001). Focus groups and individual interviews were used to encourage female interviewees to openly discuss issues related to biosphere reserves. In addition, existing reports (Huber 2011; Huber et al. 2014; Regionalverband Lungau 2011; Nigmann et al. 2016) and biosphere reserve websites have been consulted for background information as well as an overview of participatory events.

The total number of interviewees in our study is 75. We consulted 25 elderly women (60+) and 40 youth (20 years old and younger, with an almost equal male/female ratio), whereby we have adopted the definition of age groups used in intergenerational literature cited above, and compatible with the official Austrian definition of youth (aged 14 to 19 years) (Großegger, 2003). Additionally, five middle-aged women (40–50), who acted as key informants and facilitated the involvement of the elderly women, were interviewed about their perceptions of the elderly women. Furthermore, three managers (1 female, 2 male) and two process facilitators (both male) were consulted (Table 1 provides an overview of all interviewees).

**Table 1:** Overview of the interviewees

Interviewees^*a*^ and methods	Lungau	Nockberge
Process facilitators	One semi-structured qualitative interviews (male) (L-PF)	One semi-structured qualitative interview (male) (N-PF)
Biosphere reserve managers	Two semi-structured qualitative interviews with 2 consecutive managers (1 female, 1 male) (L-M, L-M2)	One semi-structured qualitative interview (male) (N-M)
Women—Interviews	3 interviews (60+) (L-E-1, L-E-2, L-E-3)	Four interviews (60+) (N-E-1, N-E-4, N-E-5)^*b*^ Four interviews (40–50) (N-E-2, N-E-3, N-E-6)^*b*^
Women—Focus groups^*c*^	1st focus group: 9 Women (60+) (L-E-5) 2nd focus group: 3 Women (60+), 1 Woman (50+) (L-E-4)	Six Women (60+) (N-E-8)
Youth—Interviews	Three interviews (16–19); 2 female, 1 male; (L-Y-1, L-Y-2, L-Y-3)	Three interviews (17–18); 2 male, 1 female; (N-Y-1, N-Y-2, N-Y-3)
Youth—World-Café	One school class, 18 pupils (16–17) (L-Y-4)	One school class, 16 pupils (13–14) (N-Y-4)
Total numbers	Forty interviewees: ∙ 16 women (3 interviews) ∙ 21 youth (3 interviews) ∙ 2 biosphere reserve managers ∙ 1 process facilitator	Thirty-five interviewees: ∙ 14 women (8 interviews) ∙ 19 youth (3 interviews) ∙ 1 biosphere reserve manager ∙ 1 process facilitator

^*a*^The age of the participants and the code names of the interviews are indicated in the parenthesis. The code names stand for: *N*–Nockberge, *L*–Lungau, *Y*–youth, *E*–elderly women, *M*–manager, *PF*–process facilitator

^*b*^Two of the interviews were conducted with two interviewees at the same time

^*c*^Participants of interviews and focus group discussion were different people

We specifically focused on women in the elderly population group due to the fact that they have been underrepresented during the participatory events and also due to the limited resources available for data collection. This approach was consulted with and agreed to by the biosphere reserve managers.

Interviews with the youth and the elderly/middle-aged women focused on information about and participation in biosphere reserve activities, their experiences with and perspectives on intergenerational activities, knowledge and skills they could/wanted to exchange between generations and the motivations for and obstacles to participation in the biosphere reserve and intergenerational practice. Process facilitators provided information on the participatory process during the reserve’s implementation. Managers were asked about the participatory process as well as their perspective on/experience with intergenerational activities relevant to the reserve. In all cases, the main questions were openly discussed first, followed by addressing specific issues on the basis of literature and previously collected data. Most interviews were recorded and transcribed (three elderly women preferred not to be recorded; the interviews with one youth and one manager were done via phone). Notes were taken by the interviewers on all occasions. During the World Café, the discussion was reflected on poster paper by both the researchers and the participants.

The data was analyzed via qualitative content analysis (Mayring 2014) using MAXQDA software, implemented separately for each case study area by applying deductive codes, followed by inductive codes. The results in each province were compared with regard to the same guiding questions (Yin 2009). Reference codes based on the province and interviewees’ age have been assigned to each interview (see Table 1) and used for citations of participants’ quotes selected for the results section. Unless stated otherwise, the results presented below are based on opinions expressed by several (3 or more) interviewees, and the citations selected represent these common opinions (although due to interaction among the participants of focus groups and World Café it is not possible to point out the exact number of interviewees agreeing with each statement).

## Results

The results are presented jointly for both biosphere reserve provinces and distinguished between them only where relevant.

### Biosphere Reserve Perception and Participation

#### Perceptions of the biosphere reserve

The 60+ women in both provinces seemed to be more skeptical with respect to the biosphere reserve than the youth, but also displayed more interest in discussing it. General associations with the biosphere reserve voiced by all interviewees were in line with the overall biosphere reserve aims: nature, agriculture, and sustainable development. However, the potential for local economic benefits was often brought up by the elderly interviewees as the key purpose of the biosphere reserve status for the region. They also expressed skepticism and frustration with the lack of the expected economic benefits from the reserve to-date. Elderly interviewees in both provinces seemed reluctant to accept the idea that the BR administration was representing the region. Many associated the biosphere reserve with restrictions rather than benefits (at least not accessible to all):

“No money became available. There is dissatisfaction” (L-E-4).

“Our farm lies within the park. The park does not bring anything to us, only to those down there, the business and tourism sector, who dominate that” (N-E-6).

Young and elderly interviewees in both provinces did not seem to have a clear understanding about the biosphere reserve function and their own potential role in it. In their view, participatory campaigns implemented in both provinces during the process of biosphere reserve designation were carried out without paying attention to the special needs of the young and the elderly population of the region. This resulted in the lack of appeal, as well as unclear information, misunderstanding, disinterest, frustration and disappointment with respect to the biosphere reserve among these groups. In Nockberge, representatives of both generations expressed confusion about the relationship between the former national park and the biosphere reserve.

#### Obstacles to participation

The interviewed elderly women in both provinces perceived the public events to be dominated by the same people, characterized by hierarchical roles and perceptions. They felt a lack of open discussion, and that the process was sometimes challenged by competition and frictions:

[The terms] “Biosphere reserve” and “management” convey that they are above us (hierarchy); it would be better to use “intermediary, transmitter” (L-E-4).

“There is no participation” (N-E-8).

Young people expressed the feeling that events and processes in the region, including those related to the biosphere reserve, are not open to participation of the youth. They also felt the lack of respect from the authorities and reluctance to accept the youth’s ideas:

“…in general, for the youth in the Lungau region, in case of a few issues […], regarding the things done in the Lungau region - we cannot share our opinion or are not asked at all” (L-Y-3).

“…if there is a person who would be open to the youth, then we would talk…” (N-Y-3).

Youth in Nockberge perceived information material about the BR as unappealing:

“The youth does not look at it. Perhaps the elderly look at it, but the youth does not look at it. Advertisement simply needs to get much better” (N-Y-3).

“If it existed in an app-format, if it would be available for a mobile phone, some people would for sure [look at it]” (N-Y-3).

Some of the elderly interviewees expressed personal reasons for the lack of involvement: old age and poor health, reservation and lack of self-confidence, lack of mobility and inconvenience. Some of the young and elderly interviewees expressed the lack of interest, as well as the lack of time for engagement, and pointed out the abundance of events and projects in the region:

“There are always so many events; there is always so much to do” (L-Y-2).

“There are a lot of events, club activities, almost an overabundance” (N-E-8).

The statements presented above imply a number of obstacles to participation by the elderly women and youth in the biosphere reserve activities, assembled in Table 2.

**Table 2:** Obstacles to and motivations for participation in biosphere reserve-related activities expressed by the youth and elderly women in Lungau and Nockberge

Obstacles	Expressed by youth	Expressed by the elderly women
Lack of benefits	-	Lack of economic benefits
	-	Frustration, negative perception, and doubts about the biosphere reserve: ∙ Aimlessness, lack of added value/benefits (L)^*a*^ ∙ Wrong handling (N)
Lack of information	∙ Lack of information, unappealing information materials, vague idea about the biosphere reserve ∙ Conceptual ambiguity with previous National Park (N)	
Obstacles related to power, hierarchy, and conflicts	∙ Power inequalities and hierarchy ∙ Lack of agreement (N) ∙ One-sided representation or perception of stakeholders (L-Y, N-E) ∙ Missing trust	
	∙ Adversarial social relationships ∙ Missing respect ∙ No addressing of youth	∙ Reservation, wariness, closeness, and lack of self-confidence ∙ Lack of feeling responsible for the reserve due to old age (N)
Personal obstacles	∙ Disinterest (N-E) ∙ Lack of time ∙ Overabundance of events in the region	
	∙ “Wrong” type of event (boring event, strict atmosphere, “wrong” participants) (N)	∙ Age-related non-participation (illness and need for care, lack of mobility, comprehension problems) ∙ Inconvenience (N) ∙ Traditional behavior patterns and gender roles (N)
Motivations	Expressed by youth	Expressed by the elderly women
Co-management	∙ Open mutual exchange on eye-level ∙ Getting involved and providing a contribution ∙ Learning and education (N)	
Benefits for community	∙ Quality and marketing of biological products, opening of a farm (N)	∙ Promoting biological product and local farmers (N) ∙ Funding (L) ∙ Consideration of duties in agricultural production, such as harvest and feeding times, when planning an event
Personal incentives	∙ Health (L-E, N-Y) ∙ Social gathering, meeting friends/peers, interest of other peers ∙ Entertainment (combination with culture/music) ∙ Personal interest in nature (L-Y) ∙ Learning and education (N)	
	∙ Convenient and relaxed atmosphere (N) ∙ Free food ∙ Money (N) ∙ Invitations via schools or associations	∙ Relevance of the topic and interest in the theme (N) ∙ Escaping loneliness ∙ Escaping routine (N) ∙ Personal invitations

^*a*^No codes indicate that the topic was raised in both areas by both age groups; codes indicate the single area, where it was raised or if it was raised by one of the age groups: *N*–Nockberge, *L*–Lungau, *Y*–youth, *E*–elderly women

#### Motivations for engagement

Many interviewed young people highlighted the importance of intangible incentives in order to encourage their participation, such as the possibility of learning something, having a good time, meeting friends, but also making a contribution, having a voice, taking part in a participatory discussion and exchanging ideas:

“I think it’s actually good that we can also give a contribution when we start such a project—either related to sports or tourism—that we simply can contribute a little bit, and we can just share our opinion, so that people become aware that young people also want to join in and are interested” (L-Y-3).

“One should just be able to exchange ideas well with others and also accept other ideas, negotiate how [the result] should look. One should in any case not oppose, reject any ideas, before listening” (N-Y-2).

Some expressed personal interests in nature and mentioned health benefits from living in the biosphere reserve. Others suggested providing tangible incentives for the youth to participate in processes, such as free food or a little bit of money. Interest of other peers in the issue has also been mentioned as a potential motivation for participation. From the perspective of the youth, BR-related events should provide entertainment value (e.g., by combining them with culture/music), allow for social learning and facilitate knowledge exchange on eye-level.

Likewise, the elderly women called for the possibility to get involved and for an open bilateral exchange. The possibilities of learning something, meeting peers, cultivating relationships, escaping loneliness, and experiencing entertainment are also of high relevance for them, as well as the potential for local economic development. While the youth prefer being contacted via institutional structures (associations, schools…), for the elderly women the obstacles to participation decrease when they are personally addressed. The elderly women also asked for more consideration of agricultural production duties, such as harvest and feeding times, when planning an event.

### Perceptions of the Elderly and Youth about Each Other and Spending Time Together

While both generations appeared skeptical, to some extent disappointed and even disinterested in the biosphere reserve, reflections on intergenerational practice were more positive. In both Nockberge and Lungau generations interact with each other, especially in regard to music and traditional songs. Other intergenerational activities comprise community festivals and events, such as traditional celebrations or card games as well as sports activities in which the old and the young take part together:

“…here are young and old in an alpine hut; they drink wine and the young and the old people are sitting together” (N-Y-3).

“We can meet each other outside, while playing sports, or perhaps during a hike” (N-E-8).

“We do this here. Meeting of generations—we have it already, but that’s more a celebration where old and young actually exchange […] has nothing to do with work […] just to bring together young and old for a bit” (L-E-3).

Both generations expressed appreciation for each other, the need and interest to interact with each other. The elderly appreciated receiving help and learning from the youth and just keeping connections with them:

“The positive things—when we come together—the positive things, which one can learn from the youth, which one can appreciate when one says: ‘‘Wow, I have learned this, and I liked it!” ” (N-E-1).

An elderly interviewee in Carinthia mentioned that women living with the youth become more open despite their historical traditional roles:

“But the woman was mostly at home, it is still strongly rooted in them […]. It is quite different when young people are at home […] They are a little more open because they are dealing with the young people and see: no, they are not so bad… they also like to speak and spend time among young people and also stay young in mind” (N-E-3).

The youth demonstrated an interest both in traditional knowledge and in sharing their knowledge and skills with the elderly. Their ideas of mutual learning included both natural and cultural heritage from the region, such as nature, cooking, music, and cultural knowledge, embracing traditional lifestyles:

“My grandfather, for example, when I go to the mountains with him, he knows the name of every single peak” (L-Y-3).

“He taught me mowing with the scythe […] I am very proud of it; only few can do it […]” (N-Y-2).

At the same time, some young and elderly interviewees assumed that the other generation might be less interested in intergenerational communication. The obstacles to and motivations for engaging in intergenerational practice, expressed in both provinces, are summarized in Table 3.

**Table 3:** Obstacles to and motivations for engagement in IP activities among the youth and the elderly women in the Lungau and Nockberge

Obstacles	As identified by the youth	As identified by elderly women
Perceived lack of interest Perceived generation gap	∙ Perceived lack of interest from the elderly to engage in topics of interest to the youth ∙ Prejudice/stereotypes by the elderly of the youth Lack of understanding of the value of learning from the youth among some of the elderly	∙ Perceived lack of interest from youth in elderly and traditional lifestyles ∙ Perceived lack of respect from the youth ∙ Criticism of the youth’ way of life/interest in technology ○ Perceived reservation by the youth to offer help
Intergenerational differences	∙ Lack of understanding by the elderly of youth dynamics and priorities	∙ Different interests and world views due to growing up in different times
Lack of communication	∙ Lack of communication from the elderly about their needs	∙ Lack of access to the young generation ∙ Lack of meeting spaces
Personal obstacles	∙ Lack of interest among some youth	∙ Reservation, wariness, closeness, shyness
Motivations	As identified by the youth	As identified by elderly women
Spending time together	∙ Celebrating/leisure: participating in some events, including music or hiking	∙ Maintaining contact with the youth ○ Receiving help ○ Doing sport activities together ∙ Willing to help youth (e.g., with the grandchildren)
Learning from each other	∙ About the local nature/mountains, the traditional land management, life skills, etc. ∙ Potential to interest more peers in traditional lifestyles	∙ About technology and current events
Mutual appreciation	∙ Trust and appreciation of the elderly ∙ Mutual respect ∙ Interest in changing stereotypes by the elderly when they get to know the young people	∙ Appreciation of the youth and their (political) worldview and potential to influence the region/world positively, also from an economic point
Personal benefits	∙ Increased self-esteem	∙ Reducing isolation

### Impressions of the Biosphere Reserve Managers About IP

Initially the managers in both provinces were not familiar with the concept of intergenerational practice and its potential benefits for the biosphere reserve. However, after having been explained the concept, they seemed positive about the existing and potential intergenerational exchanges in the region. Nevertheless, they very distinctively assessed the role of the BR management for promoting and implementing IP.

Both Lungau managers took an active role of the reserve management and thought of several IP-related ideas, which could be implemented by the biosphere reserve, such as providing students with homework focused on cultural or natural heritage in order to facilitate their discussion with the elderly, as well as extending existing biosphere reserve—related high school courses to the senior population. Other ideas included organizing intergenerational cooking sessions and facilitating support from the elderly in childcare. They highlighted the potential of technological skills of the youth in enhancing the media presence of the reserve, as well as in training the older farmers to use computers.

The Nockberge manager felt that it is neither needed nor appropriate for the biosphere reserve management to actively prompt IP, that intergenerational activities should not be “forced”, and, moreover, that the limitations posed by the old age should be taken into account. At the same time, he spoke about a computer course organized by the middle-aged women in Nockberge, in which representatives of the elderly generation took part, and acknowledged technological skills of the youth, which could be useful for such activities in the future. However, he believed that such initiatives as intergenerational activities or economic development ideas should come from the local population, and only then can be supported by the biosphere reserve. Thus, he sees the management role in providing support, but not leadership.

### Potential of IP for Involving Elderly Women and Youth in the Biosphere Reserve

The perceptions of both elderly women and youth about the biosphere reserve, their ideas about the other generation and interest in IP are similar in the two provinces. All the managers share similar awareness about IP, although those from Lungau seemed more enthusiastic about actively engaging elderly women and youth in reserve activities and considered a more active role of the reserve management in facilitating IP.

An important factor for fostering IP is the interest among the elderly and youth to interact with one another. Interviewees from both generations mentioned prevailing benefits of IP, showed much interest in the other generation and have reacted positively to the prospects of participating in intergenerational interactions. They showed greater interest in discussing IP activities than general biosphere reserve-related topics and events. At the same time, several of the proposed joint activities mentioned by the interviewees, such as exchange of traditional knowledge on land use practices, culture, nature, and landscapes, can be considered as core tasks of biosphere reserves. Altogether with the creative, entrepreneurial and technical skills of the youth, these can be further developed into innovative, place-based goods and services. Thus, based on the interviews, IP seems to hold much potential for the biosphere reserve and the fruitful participation of the younger and older generations in it.

## Discussion

### Reflection on the Research Process

#### Complementing previous studies on the reserve

The results presented in this paper with respect to the obstacles to participation in biosphere reserve activities are complementary to those found in the study of the Nockberge (Huber et al. 2014) and that of the Lungau, cited above (Huber and Arnberger 2016); following-up on previous assessments after the reserve implementation, we provide additional insights into the attitudes and needs of elderly women and youth and thus focus on the groups, which have hardly been previously approached. The frustration and negative attitudes expressed by the women and youth during the case study could be explained by the unfulfilled expectations for the biosphere reserve (Huber and Arnberger 2016), namely, of economic benefits for the local communities. The decreased enthusiasm about the Lungau and Nockberge biosphere reserve among the local residents, the lack of awareness about its functions, as well as the need for an improved, unbiased, and transparent cooperation among the reserve management and the locals were also emphasized by Humer-Gruber (2016), who investigated the perception of the reserve among the local farmers. Nigmann et al. (2016) reported that local interviewees in Lungau perceived a top-down approach from the biosphere reserve.

#### Complementarity with IP-related literature

Both the reported motivations for and the obstacles to IP are consistent with the studies and literature from other application contexts (Zeldin et al 2005; EAGLE Project 2008; Newman and Hatton-Yeo 2008; Springate et al. 2008; Pinto 2009; Van Vliet 2011), but also demonstrate some similarities between the two age groups analyzed in this study (Table 3). The motivations expressed by both the elderly and the youth: mutual learning, spending time together, as well as mutual appreciation and personal benefits—imply that IP activities could indeed attract both age groups and foster intergenerational learning. At the same time, some of the obstacles seem to result from the lack of interaction between these groups. Existing IP studies and reports refer to the origins of intergenerational biases (Vegeris and Campbell-Barr 2007) and demonstrate that the obstacles can indeed be successfully overcome via IP activities (Reisig and Fees 2007; Newman and Hatton-Yeo 2008; EAGLE Project 2008; Springate et al. 2008; Buffel et al. 2014). Existing literature also provides detailed recommendations for the implementation of IP activities, such as the importance of careful planning and selection of participants (Zeldin et al. 2005; Cohen-Mansfield and Jensen 2015), as well as open discussions and expression of opinions (Springate et al. 2008).

#### Interest by the local population

Finding interested interviewees in Nockberge was more challenging than in Lungau, which could be attributed to the negative attitudes of some landowners in this province towards the biosphere reserve, inferred by the researchers during some of the interviews. Another possible explanation is that the communities in Lungau are located in the middle of the biosphere reserve with a long-standing common identity and place-attachment, while the settlements in the Nockberge are located on the periphery of the reserve and are oriented towards several different regions only partly overlapping with the reserve. The Carinthian population may hence have a weaker place-attachment to the reserve area and may, therefore, be less interested in being engaged in biosphere reserve—related activities (Huber and Arnberger 2016).

### Integrating Solutions from the IP Field into Biosphere Reserve Management

We assume that many of the obstacles inferred from the interviews and outlined in Table 3 could be addressed by intergenerational practice, although its application in nature protection has not been investigated to-date. Based on the analysis of our results and supported by examples found in academic and practice-oriented publications from the intergenerational field, we provide suggestions of how IP-related solutions could be used to address the obstacles we have identified and improve participation of the youth and elderly women in biosphere reserve management:

#### Addressing the lack of information

The lack of understanding of and information about the biosphere reserve can be addressed directly by organizing intergenerational activities, which can be used as opportunities to inform youth and elderly about the reserve and their potential role in it. This could become the first step towards their potential participation.

In this respect, it is important to note the distinction between the abundance of information and its clarity and appeal to the underrepresented groups. The reserve managers and process facilitators provided ample examples of conducted participatory events and referred to various media (including news and online media, school presentations and discussions) used to reach and involve a broader audience in both provinces. Moreover, younger and elderly interviewees in both Lungau and Nockberge also attested to having heard about the reserve from various media sources, as well as to the “overabundance” of events in the region. At the same time, interviewers found information materials unappealing and unclear, while the “overabundance” of events in fact constituted an obstacle for both groups, implying fatigue and difficulty to prioritize.

Based on the results of the interviews, Snajdr (2016) recommends event formats specifically promoting the intergenerational interaction of the young and the elderly in the biosphere reserve. Integrating new formats to reach underrepresented groups will support the implementation of the *Strategic Action Area D* of the current MAB strategy “Comprehensive, modern, open and transparent communication, information and data sharing” (UNESCO 2017, p 24).

Several interviewees reflected their interest in intergenerational exchange: learning from the elderly, passing on stories, skills and knowledge to the younger generation. This intergenerational exchange could be something new, something different the biosphere reserve managers could focus on in order to attract both generations. The interest in topics related to cultural and natural heritage expressed by both elderly and young interviewees could provide an entrance point to discussion about the biosphere reserve during intergenerational activities. Moreover, some of the young and elderly interviewees expressed willingness to provide a contribution and be involved in the region, while others implied that they could become interested via their peers. Thus, engaging representatives of youth and elderly women in decision-making, organization of events and developing information material could pave a way for stronger involvement of these groups. While biosphere reserve-related literature does provide recommendations for participatory formats (i.e., Stoll-Kleemann and Welp 2008, Bouamrane 2007 and Creighton 2005 in Stoll-Kleemann and O’Riordan 2017), literature on IP cited here might contribute specific insights into intergenerational formats, supporting the transfer of traditional knowledge and intergenerational learning. Guidance and innovative methods for engaging youth and elderly in research activities on traditional knowledge transfer and intergenerational learning can also be used in biosphere reserve implementation (i.e., Buffel 2015; McQuaid et al. 2017).

#### Making benefits visible

Increased awareness about the biosphere reserve and its function can also help tackle the perceived lack of benefits from it. IP has been suggested as useful in increasing awareness about and acceptance of protected areas among both younger and older people. IP could be designed to support projects and processes leading to both economic benefits for local communities and the protection of natural and cultural heritage—such as creation of innovative goods and services by combining traditional knowledge with new ideas. For example, IP can facilitate sustainable tourism development based on the traditional knowledge of the older generations and technical skills of the youth and thus provide income possibilities and jobs for the youth (Mitrofanenko et al. 2015).

#### Avoiding power, hierarchy, and conflicts

Examples exist of IP addressing power inequalities and hierarchy, lack of agreement, one-sided representation or perception of stakeholders and missing trust. According to EAGLE Project (2008), IP can help stakeholder groups set priorities, take reflective actions and evaluate their efforts. A number of authors suggest that IP can facilitate community benefits, including enhanced community spirit, community cohesion, greater understanding, trust and interaction between groups in the community (Pain 2005; Zeldin et al. 2005; Vegeris and Campbell-Barr 2007; Newman and Hatton-Yeo 2008; Springate et al. 2008; Alcock et al. 2011, Mitrofanenko et al. 2015). Zeldin et al. (2005) indicated ensuring rights of participation in decision-making for the youth, promoting the positive development and empowerment of youth and building community and civil society as the purposes of ‘‘youth-adult’’ relationships. Mitrofanenko et al. (2015) suggest that IP can facilitate enhanced communication among stakeholders, active participation, and capacity development, as well as links between protected area management and other community development issues and institutions.

#### Addressing obstacles related to personal conditions

With respect to the expressed lack of interest and having other priorities, IP can lead to enhanced rates of volunteering, active citizenship, community development (Pain 2005; Vegeris and Campbell-Barr 2007), pro-social life values (EAGLE Project 2008), sense of ownership for the elderly and gratification for their contribution to the community (Newman and Hatton-Yeo 2008), as well as an enhanced sense of social responsibility in youth (Buffel et al. 2014). Mitrofanenko et al. (2015) provide examples of IP facilitating the exchange of conservation values and knowledge between the elderly and the youth, increased interest in conservation among the youth, engagement of the local population in biodiversity protection inside and outside protected areas, and community participation.

Linking IP with school-related activities can help overcome the lack of time expressed by the young interviewees. The existing collaborations between the reserve and the local schools show a number of synergies. Cohen-Mansfield and Jensen (2015) report benefits from the IP in schools to both participating seniors and children in the academic, social, and emotional domains. This could encourage opening the reserve school events to the elderly population. Such IP activities organized in schools could not only serve to provide information about the biosphere reserve to the young and the elderly, but also facilitate the envisioned role of the reserves in social learning and Education for Sustainable Development (Stoll-Kleemann and Welp 2008; Stoll-Kleemann and O’Riordan 2017, UNESCO 2017)

With respect to personal and age-related participation challenges, IP literature provides many suggestions. A number of studies point to the increased self-esteem and self-confidence of elderly and youth as a result of engagement in IP (Vegeris and Campbell-Barr 2007; EAGLE Project 2008; Newman and Hatton-Yeo 2008; Alcock et al. 2011; Buffel et al. 2014). For the elderly, IP can lead to increased skills and individual capacity, improved physical and mental health and well-being (Reisig and Fees 2007), extended social networks and new friendships, improved social and digital inclusion (Vegeris and Campbell-Barr 2007), increased energy, reduced likelihood of depression (Spence and Radunovich 2007), a renewed sense of worth, increased activity and mobility improvements, as well as ability to cope with vulnerabilities (Springate et al. 2008).

Thus, the biosphere reserve could facilitate the multiple benefits outlined above, both on the individual and community level by organizing and or supporting IP activities. This would not only constitute a useful contribution to community cohesion—an important (social) aspect of local sustainable development relevant for biosphere reserve implementation—but also enhance the view of the reserve as beneficial for the region among the participants of IP. The interest in IP activities expressed by both youth and elderly women interviewed should facilitate this process in the Lungau and Nockberge biosphere reserve. While the focus of the intergenerational literature is on youth and elderly as underrepresented groups, whose mutual contact should be facilitated, and while literature often refers to facilitating extra-familial connections in this respect, activities organized by the biosphere reserve should by no means limit participation of the middle-generations or entire multigenerational families.

### Active vs. Passive Management

With respect to more active vs. passive roles of the biosphere reserve management, some IP literature calls for caution in artificial facilitation in communities where intergenerational exchange functions well (EAGLE Project 2008). Moreover, improperly managed activities could have adverse outcomes (Vegeris and Campbell-Barr 2007). At the same time, Zeldin et al. acknowledge the importance of supportive organizational cultures, norms, policies, and structures for forming strong intergenerational relationships (2005), and other literature refers to and/or recommends a role for facilitators in such activities (Kaplan and Hanhardt 2003; Reisig and Fees 2007; Springate et al. 2008; Pinto 2009; Alcock et al. 2011; BJF 2011). Vegeris and Campbell-Barr propose “in-between” generations as potential facilitators (2007). In this respect, the reserve staff, in particular when considering the specific objectives of biosphere reserves as incubators for innovation, could take on the role of facilitators and use IP as occasions to actively involve the local youth and elderly in biosphere reserve activities. Moreover, biosphere reserve-related literature suggests an active management role in initiating and mediating participatory processes (Stoll-Kleemann et al. 2010; Stoll-Kleemann and O’Riordan 2017). In this respect, an active role for all protected area staff levels is also prescribed by the recent IUCN publication, listing competencies for protected area practitioners, which includes “ensuring inclusion of groups such as indigenous peoples, local minorities, young people, women, and those disadvantaged or underrepresented for various reasons”, as well as “Recognizing the diversity of individuals and groups among stakeholders and adapting communication approaches accordingly” (Appleton 2016, 109 and 76, respectively).

Taking initiative in organizing IP activities could require skills and competencies beyond those traditionally expected from biosphere reserve managers (Springate et al. 2008). IP literature provides suggestions in terms of competencies needed (Kaplan and Sanchez 2014 provide a summary and refer to a number of additional sources). However, as IP has been increasingly advocated for a number of social/community development fields, concise guidelines for organizing IP activities are readily available and could be used by the reserve managers (i.e., EAGLE Project 2008; BJF 2011,7). IP guidance aimed specifically at biosphere reserves is not available to-date, although recommendations for the management of the Lungau and Nockberge have been provided based on our results in Snajdr (2016).

Another important barrier to implementing IP is the lack of time and financial resources on behalf of biosphere reserve staff, which in reality often renders implementation of ambitious recommendations and biosphere reserve objectives impossible or at least challenging. These barriers, as well as the lack of competencies within the reserve, could be tackled by cooperating with institutions and professionals within the biosphere reserve community (such as local schools and social services) or working together with universities. Such cooperation could involve using resources of partner institutions, as well as developing cooperative grant applications, which could fund inviting external IP experts or hiring and training of IP personnel (these suggestions are in line with the *IUCN Global Competences Register*, Appleton 2016).

### Level and Generalizability of Recommendations

Mitrofanenko et al. (2015) proposed integration of IP into protected area management on several levels, including international, regional and national levels. Our results build on and complement their work by more specifically focusing on the individual biosphere reserve level.

Many of the obstacles and motivations to participation in biosphere reserve activities identified during our studies are similar to those reported in other publications, cited above. As such, we suggest that recommendations from this study proposed for the Lungau and Nockberge should also be relevant for other biosphere reserves, as well as eventually for other types of protected areas due to the role of biosphere reserves as a precursor of “viable protected areas in the future” (Schliep and Stoll-Kleemann 2010, p 218).

## Conclusions

The results suggest that participation of the youth and elderly women in biosphere reserve activities should and could be strengthened. Both groups need more information regarding the meaning and objectives of the reserve, how its functions relate to the developments in the region (including learning activities and leisure, as well as potential for economic development), and how they can take on an active role in the biosphere reserve by being involved in its implementation. However, the results also point to a number of obstacles to the involvement of these groups, which could be overcome via applying intergenerational practice.

Intergenerational practice is an emerging field aimed at promoting and facilitating the interaction of youth and elderly as well as achieving wider social benefits as a result of this interaction. This paper establishes a link between this field and that of biosphere reserve management. We provide evidence that there is much potential for intergenerational practice in the Lungau and Nockberge Biosphere Reserve region as both the young and the old interviewed residents are interested in an exchange with the other group; moreover, their ideas about the existing and potential interactions between the youth and elderly are related to the local cultural and natural heritage. As such, intergenerational activities could be easily linked with the biosphere reserve. They could be used to provide more information about the reserve, to build trust among the local population towards its management and to potentially build a connection with the reserve as a part of local identity.

The results imply that some of the youth and elderly might prioritize IP activities over conventional biosphere reserve events, as some of them expressed more enthusiasm about exchange with the other generation than about the biosphere reserve. Moreover, IP activities facilitated by the management could be perceived as another added value of the biosphere reserve for the region. Furthermore, the combination of traditional knowledge and skills of the elderly with the creativity and technical skills of the youth holds potential for new economic activities, which could provide local livelihood options for the younger population, such as through developing and promoting sustainable tourism products. The management could initiate and support IP projects in cooperation with schools, the local youth and elderly associations as part of biosphere reserve implementation in line with the recent MAB strategy and Lima Action Plan.

Due to the lack of existing empirical studies on applying IP in the context of protected areas, we propose that these activities should be monitored and complemented by evaluative research. Building on the overlapping motivations of young and elderly for intergenerational exchange highlighted by our empirical work, we suggest that future research should contribute to theory development and testing of methods used in other application contexts, such as via:

- Establishing and experimenting with intergenerational platforms and facilitation methods;
- Testing potential of such platforms and methods for intergenerational learning, empowerment and innovative potential (new business ideas based on old knowledge/skills and new technologies);
- Theory building on the evolution and intergenerational transfer of traditional (ecological) knowledge.

Results of such research efforts would also provide a useful contribution to the broader realm of protected area management as well as to the emerging field of intergenerational studies. This would further highlight the role of biosphere reserves as test-beds for innovative solutions towards sustainable development.

## Footnotes

1 We refer to traditional knowledge as defined in Convention on Biological Diversity “knowledge, innovations, and practices of indigenous and local communities embodying traditional lifestyles” (CBD, supra note 2, at art. 8j. https://www.cbd.int/traditional/intro.shtml).

2 “Biosphärenpark” is the German expression used in Austria to avoid the negative connotation of the term reserve (“Reservat” in German). We use the English term “biosphere reserve” throughout the rest of the paper.

3 Data from the tables: *Bevölkerung am 1.1.2017 nach Alter und Bundesland – Insgesamt; Bevölkerung am 1.1.2017 nach Alter und Bundesland – Frauen*.

4 Data for Nockberge (years 2001, 2015) and Lungau (year 2015) has been retrieved from information sheets for communities which compose the provinces of the Biosphere reserve: in Lungau (Göriach, Lessach, Mariapfarr, Mauterndorf, Muhr, Ramingstein, Sankt Andrä im Lungau, Sankt Margarethen im Lungau, Sankt Michael im Lungau, Tamsweg, Thomatal, Tweng, Unternberg, Weißpriach, Zederhaus; political district Tamsweg of Salzburg) and the Nockberge (Bad Kleinkirchheim, Krems in Kärnten, Radenthein und Reichenau; parts of districts Spittal a. d. Drau und Feldkirchen).

5 Data for Lungau in 2015: Data for political district Tamsweg (NUTS 3 area AT321 Lungau), to which Lungau is identical. *Tabelle B.8. Bevölkerungsstand nach Geschlecht und fünfjährigen Altersgruppen im Land Salzburg am 1.1.2015*. (Kurz 2015, 82–101)

6 We also refer to the biosphere reserve managers as “managers”. The biosphere reserve establishment process was accompanied by professional facilitators in both parts of the reserve. In the Lungau province, two consecutive managers were interviewed.

7 Further resources available on the websites of the EMIL network (http://www.emil-network.eu/resources/) and Generations United (http://www.gu.org/resources)
